# Thanks to Reviewers, *The Plant Genome*, 2023

**DOI:** 10.1002/tpg2.20472

**Published:** 2024-05-30

Maintaining the editorial standards of a scientific journal is the primary task of the journal editors. Their task is made much easier with the help of colleagues who are invited to review manuscripts. Through their critical comments and helpful suggestions, these volunteer reviewers have done much to maintain and further the quality of research reported in *The Plant Genome*. The members of *The Plant Genome* Editorial Board express their appreciation to the following individuals who reviewed one or more manuscripts during the past year. This list might not be complete due to problems in collating such information from many sources. We extend our apologies to those reviewers whose names have not been included.

Ackerman, A. J.

Adak, Alper

Adaskaveg, Jaclyn

Agarwal, Gaurav

Akhtar, Javaid

Akinwale, Richard Olutayo

Alahmad, Samir

Alok, Anshu

Angelin‐Bonnet, Olivia

Armstrong, Charles

Asfaw, Asrat

Ata, Haleem

Atanda, Sikiru Adeniyi

Attri, Komal

Baisakh, Niranjan

Bassi, Filippo Maria

Bauer, Petra

Bayer, Micha

Bedada, Girma

Betran, Javier

Biligetu, Bill

Brendel, Volker

Brock, Jordan

Bulle, Mallesham

C. V., Sameerkumar

Cao, Yunpeng

Castro, Patricia

Cattivelli, Luigi

Charles, Jean Rigaud

Chaturvedi, Palak

Chen, Huatao

Chen, Jianli

Chen, Xu

Chu, Qinjie

Cody, Jon

Contreras‐Moreira, Bruno

Cook, Jason

Corak, Keo

Covarrubias, Giovanny

Crain, Jared

D'Andrea, Karina

de Siqueira Gesteira, Gabriel

Dhliwayo, Thanda

Diepenbrock, Christine

Doligez, Agnès

Dramadri, Isaac

Duan, Cheng‐Guo

Fan, Longjiang

Fan, Zhen

Farhangi‐Abriz, Salar

Fei, Zhangjun

Feng, Jiawu

Ferrão, Luis Felipe

Filiz, Ertugrul

Finlayson, Scott

Fritsche Neto, Roberto

Gangurde, Sunil

Gao, Junxiang

Gepts, Paul

Ghatak, Arindam

Gimode, Winnie

Gioia, Tania

Goel, Shailendra

Golicz, Agnieszka

Goonetilleke, Shashi

Gopalakrishnan, Subrahmanyam

Grumet, Rebecca

Guerra, David

Guo, Weilong

Gupta, Ravi

Hale, Iago

Hamwieh, Aladdin

Hao, Chenyang

Harkess, Alex

Harris‐Schultz, Karen

Hayes, Chad

Hernandez, Christopher

Himmelbauer, Heinz

Hoyos‐Villegas, Valerio

Hudson, Matthew E.

Hui, Jerome

Hussain, Waseem

Innes, Roger

Jamann, Tiffany

Janga, Madhusudhana

Jegadeesan, Ramalingam

Jia, Haiyan

Jiao, Yinping

Jokipii‐Lukkari, Soile

Kane, Nolan

Kantar, Michael

Keilwagen, Jens

Kick, Daniel

Kiggundu, Andrew

Kim, Sung Ryul

Klymiuk, Valentyna

Kumar, Anil

Kumar, Jitendra

Kumawat, Surbhi

Lesueur, Didier

Leybourne, Daniel

Li, Dongdong

Li, Huang

Li, Huihui

Li, Jing

Li, Libei

Li, Zhenhua

Liao, Xiaofang

Liu, Liezhao

Liu, Shuyu

Lo, Sassoum

Lorenz, Aaron

Lubanga, Nelson

Luo, Xiao

Lysak, Martin

Ma, Jianxin

Ma, Xiongfeng

Maccaferri, Marco

Mahalingam, Ramamurthy

Mahelka, Vaclav

Maldonado dos Santos, Joao Vitor

Mandal, Swarupa

Mandel, Jennifer

Mandizvo, Takudzwa

Mandlik, Rushil

Martinez Gomez, Pedro

Mascher, Martin

Mastrangelo, Anna Maria

Mbinda, Wilton

Montardit‐Tarda, Francesc

Morota, Gota

Motte, Hans

Mourad, Amira

Mu, Qi

Mural, Ravi

Nagarajan, Ragupathi

Nahla, Bassil

N'Diaye, Amidou

Neeraja, C. N.

Nie, Xiaojun

Odeny, Damaris

Odipio, John

Ongom, Patrick

Ortiz, Rodomiro

Osorno, Juan M.

Paliwal, Rajneesh

Pandey, Girdhar K.

Pandey, Jeewan

Parida, Asish Kumar

Parker, Kyle

Parker, Travis

Pazhamala, Lekha

Pepper, Alan

Perez Lopez, Edel

Porch, Timothy

Pucker, Boas

Rahman, Habibur

Rajcan, Istvan

Raman, Harsh

ramasamy, Perumal

Ranjana, Bhattacharjee

Rauf, Yahya

Rebetzke, Greg

Rossini, Laura

Rouached, Hatem

Rouard, Mathieu

Runcie, Daniel

Sabot, Francois

Sallam, Ahmad

Salvo, Stella

Sanchez, Jacobo

Sardos, Julie

Sargent, Daniel

Saripalli, Gautam

Scarano, Aurelia.

schierenbeck, Matias

Schießl‐Weidenweber, Sarah

Schilbert, Hanna

Schmid, Karl

Schubert, Ingo

Scossa, Federico

Sharma, Gunjan

Sheehan, Moira

Shi, Haitao

Shikari, Asif

Shimelis, Hussein

Simionato, Ane

Singh, Anupam

Singh, Asheesh

Singh, Vijaya

Sirisomboon, Panmanas

Sodedji, Frejus

Sonah, Humira

Song, Qijian

Sousa Sampaio, Pedro

Spannagl, Manuel

Stahl, Andreas

Stewart, Phillip

Struik, Paul

Studer, Anthony

Stupar, Robert

Su, Erzheng

Su, Shihao

Subudhi, Prasanta

Sukumaran, Sivakumar

Sun, Bo

Suravajhala, Prashanth

Tao, Yongfu

Taranto, Francesca

Tiwari, Vijay

Tuberosa, Roberto

Uhdre, Renan

Underwood, William

Urrea, Carlos

Varotto, Serena

Vatamaniuk, Olena K.

Verma, Sujeet

Vieira, Maria Lucia Carneiro

Volpi, Nathalia

Vuong, Tri

wang, feng

Wang, Sufei

Wiesner‐Hanks, Tyr

Winn, Zachary

Wu, Di

Wungsintaweekul, Juraithip

Xia, Hui

Xiang, Yan

Xing, Chaozhu

Xu, Gen

Xu, Pei

Xu, Shizhong

Xu, Yuzhou

Yadav, Om Parkash

Yadav, Seema

Yaish, Mahmoud W.

Yamada, Yasuyuki

Yao, Yingyin

Yerka, Melinda

Yuefeng, Ruan

Zhang, Junli

Zhang, Liangsheng

Zhang, Ren‐Gang

Zhang, Runxuan

Zhang, Shoudong

Zhang, Shuang

Zhang, Wentao

Zhao, Jietang

Zhao, Peng

Zhao, Pengfei

Zhao, Yusheng

Zheng, Chaozhi

Zhou, Qiuzhong

Zhou, Yan

Zhuang, Weijian

Zikhali, Meluleki

Zvobgo, Gerald

